# Ballooning to Success: A Novel and Reproducible Sengstaken-Blakemore Model for Educating Emergency Medicine Trainees

**DOI:** 10.7759/cureus.110055

**Published:** 2026-06-01

**Authors:** David Poloway, Joel Aguilar, Suji Cha, Richard Shin, Anika Nichlany, Anita Lui, Sheetal Sheth, Rozalyn Hesse, Catherine De Guzman, Kallie Combs, Dave Simon

**Affiliations:** 1 Emergency Medicine, New York Presbyterian Queens, Flushing, USA

**Keywords:** acute gastrointestinal bleed, emergency medicine resident, emergency medicine training, hemorrhage control training, simulation design, simulation in medical education, simulation model

## Abstract

Balloon tamponade placement for variceal hemorrhage is a critical but rarely performed emergency procedure. Current hands-on low-fidelity simulation task trainers are limited, leaving a gap in high-quality training. We developed an anatomically accurate, cost-effective task trainer constructed from commonly available Emergency Department items to teach balloon tamponade device insertion for controlling variceal bleeding. This descriptive report of a low-fidelity simulation model uses 5-inch pieces of flexible silicone tubing to simulate the esophagus, connected to a 1-liter bottle as the stomach, with a 14-gauge angiocatheter inserted into the tubing to mimic variceal bleeding. The model was designed for integration with upper airway models for realistic simulation. This reproducible, cost-effective platform ($2.25 per model) provides hands-on training in balloon tamponade placement, with transparent design allowing clear visualization of device placement and balloon inflation, while modular construction enables integration with the existing simulation equipment. This innovative task trainer addresses the limited exposure to balloon tamponade procedures in emergency medicine training, providing an accessible and practical solution for skill development in managing variceal bleeding.

## Introduction

Esophageal variceal bleeding is a life-threatening complication of portal hypertension with in-hospital mortality rates up to 15%[1]. While endoscopic band ligation combined with vasoactive medications represents first-line therapy, balloon tamponade devices such as the Sengstaken-Blakemore tube serve as critical temporizing measures when initial treatments fail to control hemorrhage [2,3]. Balloon tamponade is recommended as a "bridge" to definitive therapy in patients with massive or refractory esophageal variceal bleeding, with the ability to achieve primary hemostasis in over 90% of cases [4]. However, the procedure must be performed correctly, as balloon tamponade should not be maintained for more than 24 hours and is associated with potential serious complications, including esophageal perforation, aspiration, and mucosal trauma [2,4].

Despite its critical importance, many emergency medicine trainees do not gain adequate experience with balloon tamponade due to its rare occurrence and procedural complexity [5]. Simulation-based medical education has proven to be an effective instructional strategy for both procedural and clinical skills in emergency medicine [6]. Meta-analyses demonstrate that simulation training is consistently associated with large positive effects on both process skills and product skills, with competency-based approaches showing particular effectiveness [7,8]. High-fidelity simulation programs have been shown to improve both technical and non-technical skills among emergency medicine residents, and most emergency medicine residency programs now dedicate significant resources to simulation education [6,9].

This paper describes the development of a task trainer designed to teach the procedure of insertion of a balloon tamponade device to control variceal bleeding. The model is cost-effective and constructed from commonly available Emergency Department items. This reproducible task trainer aims to enhance the education of a high-acuity, low-occurrence procedure, providing hands-on training opportunities. It addresses the limited exposure to this critical skill in the training environment.

## Technical report

Materials

The task trainer is constructed from readily available materials in any Emergency Department. These readily available materials include an emptied 1-liter saline plastic bottle, a 14-gauge angiocatheter, intravenous tubing, and suturing material. The only part that was purchased was a 3.3-foot length of flexible silicone tubing (inner diameter 1 inch, outer diameter 1 7/32 inches, $18.79). This silicone tubing was cut into 5-inch pieces, bringing the total cost of each model to about $2.25. An optional upper airway model can be connected to the model to enhance fidelity.

**Figure 1:**
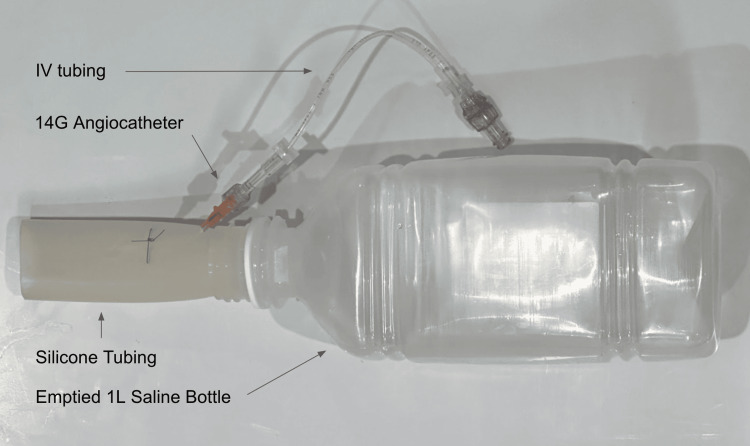
Photo of the completed model

Construction and usage

The flexible silicone tubing was cut to about 5 inches and used to simulate the esophagus. The angiocather was introduced into the lower third of the silicone tubing at a 45-degree angle, facing toward the upper part of the silicone tubing, as can be seen in Figure 1. The angiocatheter needle was retracted, with the catheter remaining in place. The angiocatheter was sutured to the tubing to secure it in place while still facilitating the passage of the balloon tamponade device during the simulation. Intravenous (IV) tubing was then connected to the angiocatheter. The silicone tubing could then be easily passed into the inner flange of the emptied bottle of saline with no securement with glue or other materials necessary. The assembly takes approximately 20 minutes. The silicone tubing can then be connected to an upper airway model for integration, requiring no additional securement for realistic simulation. During usage, red fluid (water with red dye) can then be introduced by using a simple 10 mL syringe into the IV tubing to simulate esophageal bleeding. Red fluid can also be instilled into the bottle. Fluid is easily drained by disconnecting the silicone tubing and pouring the fluid into a basin or sink. The model can be rinsed with normal tap water. The placement steps of a Blakemore-Sengstaken balloon tamponade device using the completed model are shown in Video 1.

**Video 1:** Video demonstrating use of the model simulating balloon tamponade device insertion with optional upper airway model

## Discussion

Balloon tamponade of variceal bleeding is a critical procedure for managing life-threatening esophageal variceal bleeding. However, many emergency medicine trainees do not gain adequate experience due to its rare occurrence [5]. The procedure requires precise technical skills, including proper tube placement, sequential balloon inflation, and appropriate traction application. Complications from improper technique can be severe, with historical studies reporting serious adverse events in up to 47% of cases with balloon tamponade, including esophageal perforation, aspiration pneumonia, and airway obstruction [4,10]. As a result, there is a need for an affordable, reproducible simulation task trainer that allows trainees to practice this life-saving intervention in a controlled environment.

This innovative model presents a cost-effective, easily reproducible trainer for experiential learning of balloon tamponade for variceal bleeding such as a Sengstaken-Blakemore tube. It can be constructed from readily available materials, making it accessible for residency programs with limited resources. It consists of an emptied 1-liter bottle of saline to simulate the stomach and flexible silicone tubing to represent the esophagus. A 14-gauge angiocatheter is inserted to simulate variceal bleeding, with red fluid delivered through intravenous tubing to enhance realism. During multiple uses, no leakage of fluid was observed in the model. This is likely due to the flexible silicone tubing creating an expanding seal around the angiocatheter insertion site and the direct fit between both the upper airway model and the emptied bottle of saline. The simple design allows for quick assembly and deliberate practice, helping emergency medicine trainees develop motor and cognitive skills to successfully perform this procedure.

Additionally, the modular design of this model allows for customization, enabling integration with other airway or gastrointestinal models for interdisciplinary training. The model can be connected to a mannequin's esophagus to practice the placement of a balloon tamponade device through the upper oropharynx, adding another layer of realism. This integration capability is particularly valuable given that balloon tamponade placement often requires concurrent airway management, as patients with massive variceal bleeding frequently require endotracheal intubation prior to device placement [11].

The transparent design of the silicone tubing provides a unique educational advantage by allowing direct visualization of the balloon tamponade device during insertion and inflation. This feature enables learners to understand the spatial relationship between the gastric and esophageal balloons, the esophageal lumen, and the simulated bleeding source. Such visualization is critical for developing the mental model necessary for a safe and effective device placement in clinical practice.

The educational value of this model is supported by extensive evidence demonstrating the effectiveness of simulation-based training for procedural skills. A systematic review and meta-analysis of procedural instruction found that simulation and competency-based approaches were the most effective forms of training for invasive bedside procedures [7]. Emergency medicine residency programs increasingly recognize the value of simulation, with 95.8% of programs reporting access to dedicated simulation centers and 90% having task trainers available [6].

Simulation-based training has been shown to improve procedural competency, reduce cognitive load, and build confidence during high-pressure situations [9,12,13]. This model offers emergency medicine trainees an opportunity to practice a critical procedure they may rarely encounter. Its transparent design, cost-effectiveness, and clear visualization could make it an indispensable tool for training programs, particularly those with limited resources, by providing an accessible and practical solution for skill development. 

This model has several inherent limitations that should be acknowledged. As a low-fidelity task trainer, it does not provide a realistic tactile feedback, particularly the resistance encountered when passing the balloon through the gastroesophageal junction. Additionally, the use of a rigid 1-liter plastic bottle to represent the stomach does not replicate the distensibility and compliance of a real human stomach, which may limit the fidelity of gastric balloon inflation. However, compared to commercially available high-fidelity simulators, which can cost thousands of dollars and may not be readily accessible in all training environments, this model offers a highly cost-effective and reproducible alternative constructs from the commonly available Emergency Department items. Its low cost and ease of assembly make it particularly well-suited for programs with limited simulation budgets. It is also important to note that no formal or informal survey data were collected from residents who used the model and, therefore, objective educational validation of this task trainer has not yet been performed.

The clinical context for this training is increasingly important. While balloon tamponade has been partially supplanted by self-expandable esophageal stents in some centers, these stents are not Food and Drug Administration (FDA)-approved in the United States, and balloon tamponade remains the standard temporizing measure for refractory variceal bleeding in most emergency departments [11]. Emergency physicians must be prepared to perform this procedure as a bridge to definitive therapies such as transjugular intrahepatic portosystemic shunt (TIPS) or endoscopy [2].

Future research should assess the impact of this task trainer on skill retention and confidence among emergency medicine trainees. Comparative studies evaluating learning outcomes between this low-fidelity model and higher-fidelity alternatives would help establish its educational effectiveness. Additionally, using this platform to practice insertion of other balloon tamponade devices such as the Minnesota or Linton-Nachlaus tube could be explored [11,14].

## Conclusions

This novel, low-fidelity simulation model provides an anatomically accurate, cost-effective, and reproducible platform for training emergency medicine residents in balloon tamponade placement for variceal hemorrhage. Constructed from readily available materials, the model can potentially address a critical gap in procedural training for a high-acuity, low-occurrence (HALO) emergency intervention. The transparent design allows for a direct visualization of device placement and balloon inflation, which has the potential to enhance the learning experience. By providing accessible hands-on training opportunities, this task trainer has the potential to improve trainee preparedness for managing life-threatening variceal bleeding and represents a valuable addition to emergency medicine simulation curricula, particularly for programs with limited resources.
